# Poultices in Pulmonary Diseases of Children

**Published:** 1897-11

**Authors:** J. M. G. Carter

**Affiliations:** Professor of Clinical and Preventive Medicine in the College of Physicians and Surgeons of Chicago; President of the Illinois State Medical Society, etc.


### POULTICES IN PULMONARY DISEASES OF CHILDREN.

BY J. M. G. CARTER, M. D., Sc.D., Ph.D.

Professor of Clinical and Preventive Medicine in the College of Physicians and Surgeons of Chicago; President of the Illinois State Medical Society, etc.

This important but common subject would not need discussion now were it not that the principles of therapeutics, represented by the proper use of poultices, seem to be forgotten at times or their operations rejected. When professional men like Senior (*British Medical Journal*, April 3, 1897) and others eminent in the domain of medicine object to the poultice because "it gets cold when allowed to remain on several hours," and thus becomes an exciting cause of further disease or a relapse, as changing from a warm to a cold room might, it is necessary for some one to remind these critics of the purpose for which a poultice is used, and the method of its application. The poultice may be used to relieve pain, or to produce *local* dilatation of the capillaries to relieve some congested area. Now, if these are the objects sought, this is certainly one of the agents which may be used, especially in the pulmonary diseases of children. The *method of use* is as important in the employment of this remedy as in the case of any other. It would be useless in many instances to look for good results from the administration of a dose of quinine once a week or even once a day. It is just as unwise to expect decided effects from a poultice when used in a haphazard or irregular way or at great intervals. Directions must be definite and results will be positive.

In broncho pneumonia, pneumonia, or bronchitis, it is not always advisable, but is often beneficial. If the patient is poor, the apartment cold, and provisions for nursing meager, it may be better not to undertake the use of this agent. The method and circumstances are everything. Under favorable conditions—that is, under conditions where the physician's directions can be faithfully carried out—the poultice is of great value. When directions can not be fully conformed to this agent should not be used—a rule which applies to all remedies.

In broncho-pneumonia, when dyspnea is marked; in pneumonia, when pain is great; in pleurisy and bronchitis, accompanied by much distress—a hot poultice surrounding the body will often give very quick and permanent relief; but it must be kept hot by frequent changing or covering with oiled silk and heating with hot-water bottles.

The failure to get good results from the use of poultices indicates a failure to observe therapeutic indications, or negligence in the method of using the remedy. The fault, then, is not with the poultice as a remedial agent, but with the method of use, or with the using it when there is some contraindication. It is no more a panacea for all pulmonary ailments than is Dover's powder. A differential study of cases will help to determine the conditions in which this remedy is of greatest value, and will aid in restoring the poultice to its former deservedly high place in the minds of the profession, and conduce to a proper limitation of its field of usefulness. Exact therapeusis requires that a remedy be prescribed at the proper time, in a definite manner, for a known purpose. Other important local applications which require as careful and scientific use are the mustard, capsicum and spice poultice or plaster; stupes of turpentine, camphor, alcohol; jackets of spongiopiline, cotton, lamb's wool. None of these can be applied in every case indiscriminately.—*Medicine.*

